# The Truth Plainly, Forcibly Written

**Published:** 1871-10

**Authors:** 


### THE TRUTH, PLAINLY, FORCEIBLY WRITTEN.

I am greatly obliged for a copy of the History of the Controversy between the Faculty of the Atlanta Medical College, the Board of Trustees and the Medical Association of the State of Georgia. I have read it attentively, with the view of arriving at a just conclusion upon the subject; and must confess that I have never read a more satisfactory argument in my life. It even unravels and presents to the gaze of the public, the hidden mysteries which have heretofore been covered up. Truth may be crushed, yet, at last, it will rise brilliantly above the clouds which overshadowed it.

My son having graduated in 1859, in the Atlanta Medical College, awakened a deeper interest in reading the Report, because I had heard that the old Board of Trustees had treated the Faculty with great injustice; and that if their views were successfully carried out, would have the effect to invalidate or destroy the legality of all the Diplomas after the passage of the amendment, in 1858; for the reason, that the original charter was imperfect, and the amendment was absolutely necessary, and that all who graduated after the passage of the amendment, up to the close of the session of 1868, received their Diplomas under the amendment—that Dr. Powell and the old Board acquiesced in conducting the College for 8 years under the amendment, and then united in their efforts to destroy the College and disgrace its graduates. Hearing these things, I, as well as my son, feared that Dr. P. and the Trustees were wrong in their positions and efforts; but since reading the history of the difficulty, prepared and published by the Fulton County Medical Society, we have become entirely satisfied that Dr. P. and the old Board of Trustees were right—they could have taken no other position, and been sustained by the laws of the State and ethics of the Profession. The facts also set forth in this history, proves, beyond a doubt, that their positions alone can save the Institution and protect the rights and character of its graduates from its commencement up to 1866.

As your report states, if the original charter was imperfect, then there never was a legal lecture delivered in the Institution, or a legal Diploma issued. It also, proves that neither Dr. P. nor the old Board ever acquiesced in or acted under the amendment.

It shows that the Fulton County Medical Society simply reported to the Georgia Medical Association, which met in Augusta, the condition of the College, as reported by the Board of Trustees. The report of the Georgia Medical Association, in Savannah, shows that the meeting at Augusta, in 1868, did nothing but endorsed the action of the Board of Trustees—could it have done less after the Board had declared it to be illegal and refused to recognise it? The action in Augusta was not against the Faculty as a body, or individually, but against the College as then organized! Could the Association in Savannah, in 1869, have done less? It declared the charges made against the Association, by the Faculty, to be wilfully false. But did they expell them? No; but gave them another year to retract· Did they retract? The Association, which met in Macon in 1870, thought not; and in justice to the members of the Association, who met in Augusta in 1868, and in Savannah 1869, expelled the Faculty. Could they have done less? The Medical Association, at Macon, thought not. At the last session of the Association, held in Americus, these three last actions by the Association, were declared to have been from personal feeling, and thereby expunged from the minutes, which I considered, and think every unbiassed man will unite with me in the same conclusion, was a greater slander, if possible, than the charges preferred against the Association which met in 1868. I think that this last action, at Americus, should be freely discussed for the benefit of all concerned. I do not belong to that classs who believe that controversies measurably do harm. Free and full discussion is often absolutely necessary in order to arrive at the truth, that peace may be established. It is not being engaged in a heated controversy that injures an individual, but, in my judgment, it is the positions taken and the manner and means used to maintain these positions—therefore, I am for continuing the investigation until it is settled upon the basis of truth and justice. I am now told that the apology made at Macon was ample and full, and ought to have been received, and that that apology was suppressed, and never published for the benefit of all. I hope you, gentlemen, will publish the charge and the apology, and let your readers judge for themselves. My son will address Dr. Powell soon, he now agrees with him and the old Board in the opinion that the original charter needed no change, and that the Diplomas issued up to '66, are legal and its graduates regular; he also, makes this point, that the regular graduates of the Institution should not recognize those who graduated under the Amendment, if it were made known by the agrieved party. It strikes me that the difficulty gets worse and worse. For the resolution passed in Americus certainly degrades the Profession more than the charges made in 1868.

---

The above extract we take from the letter of an old and esteemed professional gentleman of this State. Being a private letter, we withhold the name.

The positions are well taken. They are true. Our correspondent fortifies them by an appeal to truth; and he sustains the truth thus appealed to, by a most thorough and convincing argument. No cause—no controversy—ever presented an array of more overwhelming and incontrovertable facts to sustain the high professional course of the Georgia Medical Association in its actions against the old Faculty of the Atlanta Medical College.

Facts are imperishable. No good man can *long* afford to stand by and assist in the overthrow of principle. Sooner or later the truth will triumph, bringing honor to its advocates and supporters, and shame and confusion to its enemies and opposers. We sincerly beg good men to investigate these facts—we also, beg those who oppose principle, to be warned in time, before they endorse error and become as guilty as those who seek to cover up the truth and if possible, to overthrow it.

